# Consonant Proficiency From 18 Months to 10 Years in Swedish Children With Unilateral Cleft Lip and Palate

**DOI:** 10.1111/1460-6984.70338

**Published:** 2026-09-15

**Authors:** Anette Lohmander, Inghild Riis Kjelsvik, Christina Persson

**Affiliations:** ^1^ Division of Speech and Language Pathology, Department of Clinical Science, Intervention and Technology Karolinska Institutet Stockholm Sweden; ^2^ Department of Health and Rehabilitation, Institute of Neuroscience and Physiology, Sahlgrenska Academy University of Gothenburg Gothenburg Sweden; ^3^ Department of Otorhinolaryngology Region Västra Götaland Sahlgrenska University Hospital Gothenburg Sweden

**Keywords:** cleft palate, oronasal symptoms, percentage of consonants correct, speech development, surgical protocols

## Abstract

**Background and Aim:**

Longitudinal speech results in children born with cleft palate with or without cleft lip are sparse. The aim of this study was to investigate the longitudinal development of consonant proficiency in Swedish children born with unilateral cleft lip and palate (UCLP).

**Methods and Procedures:**

Previously collected data on Swedish children with UCLP treated in Gothenburg and Stockholm were retrieved from the Scandcleft datasets. Only children with complete data on babbling at 18 months, and speech variables based on phonetic transcription at 3, 5 and 10 years of age were included (*n* = 32). Three surgical procedures for palatal closure were represented. At both centres, half of the children had undergone a two‐stage palatal repair with soft palate closure at 6 months and hard palate closure at 12 months. The remaining children received either the same procedure but with hard palatal repair at 3 years (after the 3‐year assessment), or a one‐stage complete palatal repair at 12 months. Data were analysed using descriptive statistics on Percentage of Consonants Correct (PCC) and on oronasal symptoms at 3, 5, and 10 years of age, and for PCC also with correlation and regression analyses to examine the predictive value of early consonant production.

**Outcomes and Results:**

Despite substantial individual variation, there were no significant group‐level differences in the main outcomes across primary surgical procedures, with the exception of a higher prevalence of oronasal symptoms at 3 years in the subgroup with unrepaired hard palate. Average PCC improved by age but remained below that of peers at all time points. Presence of oral stops at 18 months was the only variable that predicted a higher PCC at later ages. The percentage of oronasal symptoms decreased with age. Children who had not undergone secondary surgery and had a low number of speech therapy visits (indicating less need) exhibited the highest PCC and the lowest percentage of oronasal symptoms.

**Conclusions and Implications:**

The findings suggest that early consonant production may serve as a predictor of later consonant proficiency. The large individual variation indicates that a need for individualised speech‐language follow‐up in addition to standardised documentation, and that factors beyond the surgical protocol influence speech outcomes.

**WHAT THIS PAPER ADDS:**

*What is already known on this subject*

- Both early consonant production and later consonant proficiency in children with cleft palate with or without cleft lip have been shown to be poorer than in peers. However, the longitudinal aspect has been sparsely investigated, and true longitudinal data are needed.

*What this paper adds to existing knowledge*

- The longitudinal outcome regarding consonant proficiency support previous findings indicating a small or negligible impact of primary surgical protocol. Furthermore, although the study group was small, the findings were consistent with results from the main Scandcleft project. This was also the case for the lack of impact from secondary surgery and speech therapy visits. The individual variation was substantial. However, the presence of oral stops at 18 months was a strong predictor for consonant proficiency at later ages.

*What are the potential or actual clinical implications of this work?*

- Soft palate repair is crucial for establishing the prerequisites for oral stop production during babbling. However, also factors beyond the surgical protocol influence speech outcomes. Individualised speech‐language follow‐up in addition to standardised documentation is recommended. This should aim to identify outliers and offer parent‐implemented support to individual infants with delayed babbling.

## 1 Introduction

The primary goals in the treatment of children with cleft palate with and without cleft lip (CP±L) are to achieve optimal speech production, preferably enabling children to perform on par with their peers before school entry (Persson et al., 2025), and to achieve optimal growth of the facial skeleton (Friede et al., 2013; Ness et al., 2015). Early cleft palate surgery is important for speech development but may negatively affect maxillary growth, which has led to interest in delaying closure of the hard palate. However, while early soft palate closure is considered beneficial for velopharyngeal function, an opening in the hard palate may lead to air leakage and reduced intraoral pressure on consonants. A consequence is sometimes retracted oral articulation, when the closure for oral stops is performed behind the opening (e.g., Lohmander et al., 2012). Most studies have investigated the impact of surgical methods on children's speech at specific ages but few have examined speech development from a longitudinal perspective (Persson et al., 2025). Understanding how speech production changes over time in children with CP±L, as well as the potential influence of different surgical methods, remains an important research focus.

Some of the research on different primary surgical protocols and their effects on speech development is based on data collected in a standardised manner through the Scandcleft project (Semb et al., 2017). These data also form the basis for the present study. The Scandcleft project, initiated in 1997, was designed as a randomised controlled trial with the aim of investigating how different surgical protocols for primary cleft palate repair influence outcomes at 5 years of age. A total of 448 children with non‐syndromic complete unilateral cleft lip and palate (UCLP) were recruited from cleft centres in Sweden, Norway, Denmark, Finland and the United Kingdom. The project comprised three parallel trials comparing local surgical protocols (Arms B‐D) with a common protocol (Arm A). Both Arms A and B involve two‐stage procedures with lip and soft palate closure at 3–4 months, followed by hard palate closure at either 12 months (Arm A) or 3 years (Arm B). In contrast, Arm C involves lip closure at 4 months and a single‐stage closure of the hard and soft palate at 12 months, while Arm D consists of a two‐stage protocol with lip and hard palate closure at 4 months and soft palate repair at 12 months (Semb et al., 2017).

To evaluate the influence of these protocols on speech outcomes, perceived velopharyngeal function (VPF), hypernasality and consonant production were assessed at 5 and 10 years of age with the results reported in several publications (Lohmander, Persson, et al. 2017; Willadsen et al., 2017; Hammarström et al., 2020; Persson et al., 2020; Willadsen et al., 2023). Consonant proficiency was assessed using Percentage of Consonants Correct (PCC) (Shriberg et al., 1997; Willadsen et al., 2017). VPF was assessed using VPC‐Sum, a composite measure developed within the Scandcleft project that includes hypernasality, active non‐oral errors, and passive symptoms of velopharyngeal insufficiency (Lohmander, Persson, et al. 2017). Although not part of the main trial, additional data were collected at 11–12 months, 18 months, and 3 years of age and have been used in subprojects conducted in Sweden and Denmark (Lohmander et al., 2011; Willadsen, 2013; Klintö et al. 2014, 2016, Raud Westberg et al., 2019). Consonant production at 18 months was assessed through analysis of audio‐video recorded play sessions, and at Age 3, consonant production was phonetically transcribed and PCC adjusted for age was used as a measure of consonant proficiency.

Two of the subprojects within the Scandcleft project have shown that children who underwent early soft palate closure at approximately 6 months of age as part of a two‐stage protocol (Arms A and B) produced a high mean frequency of oral stops at 12 months of age, that is 25.5% (Willadsen & Albrechtsen, 2006) and 29% (Lohmander et al., 2011) compared with children in other studies, who underwent one‐stage palate closure at approximately 12 months of age. Hutters et al. (2001) reported a mean ‘pressure consonants’ occurrence of 2%, and Jones et al. (2003) reported 7.4%. However, Scherer et al. (2008) reported that 23% of the 13 children who had palatal surgery at 10–12 months of age used oral (velar) stops at 12 months. All assessments were performed by phonetic transcription from audio‐video recordings or audio recordings. They lasted 30–45 min, aiming for 100 child utterances, and consisted of play with parent and a fixed set of age‐appropriate toys, except for in the study by Jones et al. (2003), where the data collection was carried out at children's homes and lasted for 1–2 h. At the time of testing, children assigned to the two‐stage protocol had a repaired soft palate but an unrepaired hard palate, whereas children assigned to the one‐stage protocol had neither the soft, nor the hard palate repaired. The findings suggest that early soft palate closure may facilitate prelinguistic development. However, whether palatal closure is performed in one or two stages does not appear to influence speech outcomes in a longer perspective. In the Scandcleft subproject by Raud Westberg et al. (2019) consonant production at 12, 18 and 36 months was compared in children treated with either a one‐stage (Arm C) or two‐stage protocol (Arm A). While Arm A showed an advantage at 12 months, likely because the soft palate had already been repaired at that age, no differences were found at 18 months or 3 years. This aligns with the Scandcleft main studies and additional substudies demonstrating comparable speech outcomes at 3, 5 and 10 years between one‐ and two‐stage protocols (Willadsen, 2012; Klintö & Lohmander, 2017; Willadsen et al., 2017; Lohmander, Persson, et al. 2017; Hammarström et al., 2020; Willadsen et al., 2023; Persson et al., 2025).

Importantly, in addition to surgical timing and protocol, several other care‐related factors may influence speech outcomes, including surgeon experience, cleft width, hearing impairment, secondary surgical interventions, and speech therapy (Shaw & Semb, 2017; Sand et al., 2022; Butterworth et al., 2023; Sainsbury et al. 2024). Notably, Persson et al. (2025) found that neither secondary VPI surgery, nor number of speech therapy visits was associated with speech outcomes at 5 and 10 years.

Multiple studies both within and outside the Scandcleft framework have previously demonstrated associations between prelinguistic vocalisations and speech‐language development in toddlers with CP±L. Predictive factors include canonical babbling ratio/mean babbling length (Chapman et al., 2003; Scherer et al., 2008); production oral stops (Chapman et al., 2003; Klintö et al., 2014; Raud Westberg et al., 2019; Lohmander et al., 2021), dental/alveolar stops (Lohmander & Persson, 2008; Klintö et al., 2014; Lohmander et al., 2021), number of true consonants (Chapman et al., 2003; Lohmander et al., 2021) and number of different consonants (Lohmander & Persson, 2008; Scherer et al., 2008; Klintö et al., 2014; Raud Westberg et al., 2019). Additionally, consonant production at 11 months has been identified selective for word initial consonant at 18 months (Willadsen et al., 2013) and number of stop consonant production at 16 months as a predictor of reported expressive vocabulary at 24 months (Wilder et al., 2026).

However, several studies are based on small samples, include mixed cleft types, or apply unspecified selection criteria (Sell, 2005; Shaw et al., 2016). The large number of primary surgical protocols further complicates comparisons across studies. Although a few longitudinal studies have been conducted, most are small and report outcomes, with a single exception at two or three time points, for example as part of the Scandcleft project or its substudies (Lohmander et al., 2011; Klintö et al., 2016; Raud Westberg et al., 2019; Persson et al., 2025), as well as beyond (Hutters et al., 2001; Chapman et al., 2003; Lohmander & Persson, 2008; Scherer et al., 2008; Lohmander et al., 2012; Nyberg et al., 2014; Lohmander et al., 2021). See Appendix for brief information on sample size, cleft type, age of data collection and relevant findings. The limited total number and sample variation justify why more studies are needed.

The Scandcleft project provides a continued valuable opportunity for extended longitudinal analyses encompassing earlier developmental ages. Consequently, the aim of the present study was to investigate the longitudinal development of consonant proficiency from 18 months to 10 years of age in Swedish‐speaking children born with UCLP included in the Scandcleft project.

### 1.1 Research Questions

1. Are there differences in PCC or oronasal symptoms at 3, 5 or 10 years of age in relation to primary surgical protocol?
2. How does PCC change between 3, 5 and 10 years of age, and to what extent is it associated with consonant production at 18 months?
3. What is the prevalence of oronasal symptoms on consonants at 3, 5 and 10 years of age, and to what extent do these symptoms decrease or persist over time?
4. What impact do background variables (hearing, speech therapy visits, secondary surgery) have on speech outcomes?

## 2 Method

### 2.1 Participants

All participants in this longitudinal study were part of the Scandcleft project and treated at either Karolinska University Hospital in Stockholm or Sahlgrenska University Hospital in Gothenburg, Sweden. The recruitment process, participant flow, distribution across surgical protocols, and randomisation procedures are described in detail by Semb et al. (2017).

To be included in the Scandcleft project, the children had to be born with a non‐syndromic unilateral complete cleft lip and palate, accepting a soft tissue bridge not exceeding 5 mm. Furthermore, since the main project included evaluation of facial growth, dental development and occlusion, in addition to evaluation of speech and velopharyngeal function, the morphology of the face had to be controlled for. Therefore, being Caucasian was also an inclusion criterion. Furthermore, eligibility depended on at least one caregiver being a native speaker of the official language of the country of residence and using that language in the home. A total of 35 children treated in Stockholm and 20 children treated in Gothenburg were included. Participants in Gothenburg were randomised to either Arm A or B (Trial 1), and participants in Stockholm to either Arm A or C (Trial 2) (see Introduction), with an equal allocation to each protocol (Semb et al., 2017). For inclusion in the present study, participants also needed to have complete data for all outcome variables at 18 months, 3, 5 and 10 years of age. Of the 55 children enrolled in the Scandcleft project, 23 were excluded due to missing data. Thus, a total of 32 Swedish children met the criteria, with 18 (56%) treated in Stockholm and 14 (44%) treated in Gothenburg (Table 1).

**TABLE 1:** Background data for the participants (*n* = 32).

ID	b (boy) g (girl)	Arm^a^	PTA 3 Y (P/F)	PTA 5 Y (P/F)	Sec. Surg. < 5 Y	Sec. Surg. < 10 Y	ST < 5 Y	Total ST ≤ 10 Y
1	g	A	mv	mv	No	No	11	12
2	g	A	P	P	No	No	13	15
3	g	A	P	P	No	Yes	16	28
4	g	A	P	P	No	No	7	9
5	g	A	P	P	No	No	5	7
6	b	A	P	P	No	No	6	9
7	b	A	P	P	No	No	13	18
8	g	A	F	P	No	No	44	66
9	b	A	F	P	No	No	28	41
10	b	A	P	P	Yes	Yes	22	67
11	b	A	F	P	No	No	20	21
12	b	A	P	P	No	No	20	21
13	b	A	P	P	No	No	6	6
14	b	A	P	P	No	No	9	10
15	b	A	P	P	No	No	13	14
16	b	A	P	P	No	No	24	36
17	g	B	P	mv	No	Yes	13	17
18	g	B	P	P	No	No	16	20
19	g	B	P	P	No	No	33	55
20	g	B	P	P	No	No	7	9
21	b	B	P	P	No	No	10	16
22	b	B	P	P	No	Yes	14	38
23	b	B	F	P	No	No	7	17
24	g	C	mv	mv	No	No	5	5
25	g	C	P	P	No	No	17	18
26	b	C	F	P	No	Yes	15	22
27	b	C	P	P	No	Yes	11	45
28	b	C	F	P	No	Yes	16	21
29	b	C	P	F	No	No	16	17
30	b	C	P	P	No	Yes	27	29
31	b	C	P	P	No	Yes	17	20
32	b	C	mv	mv	No	No	5	6

^a^A = lip and soft palate closure at 4 months, hard palate closure at 12 months (common Arm).

B = lip and soft palate closure at 4 months, hard palate closure at 3 years. NB: the hard palate cleft was unoperated at the 3‐year assessment.

C = lip closure at 3 months, soft and hard palate closure at 12 months.

PTA = Pure Tone Average on the best ear; P = pass, ≤20 dB, F = fail, >20 dB, mv = missing value, Sec.Surg. = secondary velopharyngeal surgery, ST = speech therapy visits.

All parents gave written informed consent for participation. Ethical approval was obtained from the appropriate Regional Ethical Committee.

### 2.2 Material

The material consisted of a selection of data already collected as part of the Scandcleft project, which included not only the main outcome ages of 5 and 10 years but also the earlier ages of 18 months and 3 years.

#### 2.2.1 18 months

At 18 months, early consonant production was assessed using an audio‐video recorded play session lasting 45–60 min. Children interacted with a parent using a fixed set of age‐appropriate toys. An experienced speech‐language pathologist (SLP) completed pseudonymised observation forms, thus being blinded to surgical procedure, noting the presence or absence of variables related to babbling, that is oral stops, dental stops and number of different consonants. Consonants heard at least twice in two different utterances were recorded.

#### 2.2.2 3, 5 and 10 years

Speech material at 3, 5 and 10 years consisted of the elicited single words from a 33‐word test developed for the Scandcleft project (Lohmander et al., 2009). The test targeted ten consonants: six oral stops /p, b, t, d, k, g/, three fricatives /f, v, s/ and one nasal /n/. Each occurred three times in stressed initial position; additionally, /s/ also occurred three times in final position. At age 3, only words with final /s/ were elicited, as appropriate for the age (Blumenthal & Lundeborg Hammarström, 2014), yielding 30 words in total at Age 3. The words were elicited with picture stimuli and audio‐video recorded. If the child could not retrieve the target word and semantic cues were insufficient, imitation was allowed. At least 50% of the target words had to be produced for inclusion in the analyses (Klintö et al., 2014; Raud Westberg et al., 2019).

The standardised recordings took place in a quiet room at each hospital as part of the children's regular follow‐up with the cleft palate team. All elicited word productions were later transcribed using IPA (International Phonetic Association, 2015) and extIPA (International Phonetic Association, 2016). Independent transcription was performed by SLPs blinded to the surgical protocol, with no restriction on the number of times the audio recordings could be replayed. At 3 years two SLPs not involved in the project performed the transcriptions. At 5 years, four SLPs, who participated in the Scandcleft project, transcribed the recordings from a centre other than their own. One of these, also transcribed all 10‐year‐olds. Another, of the four, transcribed those from the centre she was not part of and an additional transcriber, also part of the Scandcleft project but not involved in the actual centres, transcribed the remaining. The total number of transcribers was five at 5 and 10 years.

In the analysis of percentage of consonants correct (PCC) were passive speech errors accepted as correct if the consonant was otherwise produced with the correct place and manner. The number of passive oronasal symptoms (nasal emission, nasal turbulence, weak pressure, nasal realisation of voiced oral consonants) was summarised based on the transcripts of the speech tests.

### 2.3 Reliability and Validity

During the development of the Scandcleft project and prior to the analyses at the primary 5‐year outcome age, extensive training in phonetic transcription was provided. The training sessions were held every 2 years for 2–3 days (Lohmander et al., 2009; Lohmander, Persson, et al. 2017; Willadsen et al., 2017, 2023). All audio‐video recordings were presented in random order and independently assessed by two SLPs who were blinded to the participants’ treatment history. Re‐assessments were conducted to ensure consistency.

Reliability of the babbling measures was evaluated by comparing observation data with data from phonetic transcription. During the observation, the presence of a consonant was noted when two or more productions were heard in different utterances, and from the transcription if a consonant was transcribed two or more times in different utterances. Semi‐narrow transcriptions using IPA and diacritics (IPA, 1999) of between 77 and 100 speech‐like utterances per recording containing at least one vowel‐like or consonant‐like element or combination, had been performed independently by one SLP and one 4th‐year SLP student. Before that, they had identified and edited all vocalisations using breath groups (Nathani & Oller, 2001), and excluded non‐speech‐like utterances, for example, burps, grunts and cries. Vowels were only marked. While consonant place and manner were counted, voicing errors were ignored. Intertranscriber agreement on place and manner was calculated on a randomly selected 20% of the utterances. Intratranscriber agreement was calculated from the re‐transcription of 20% of the utterances performed 1 month later. Reliability of the observations has previously been reported (Lieberman & Lohmander, 2014), and the transcriptions were calculated point by point (Lohmander et al., 2011). Good absolute agreement was found for oral stops (87%) and dental/alveolar place (79%), whereas agreement for number of different consonants was poor (24%); grouping by categories (≤5, 6–10, >10) improved agreement to 63% (Lieberman & Lohmander, 2014). Reliability of phonetic transcription at later ages varied by assessment point. At 3 years: 33% re‐transcribed; mean inter‐transcriber reliability 70% and 90% for the two centres, respectively; intra‐transcriber agreement 87% and 92% (Klintö et al., 2014; Raud Westberg et al., 2019). At 5 years: 100% of material double‐transcribed; inter‐transcriber agreement 86% and mean intra‐transcriber agreement 89%, when minor disagreements were accepted (Willadsen et al., 2017). At 10 years: 20% re‐assessed; mean inter‐transcriber agreement 91% and mean intra‐transcriber 92% of correct or incorrect realisation of the target consonants (Willadsen et al., 2023).

### 2.4 Procedure

At 18 months, variables derived from the observation forms; presence of oral stops and dental/alveolar place of articulation, and number of different consonants as an indicator of the breadth of the child's established consonant inventory, were used as predictors. Outcome variables were PCC and percentage of oronasal symptoms at 3, 5 and 10 years. Symptoms attributable to residual clefts, post‐operative fistulas and velopharyngeal insufficiency were classified as oronasal symptoms. The number of target consonant transcribed with an oronasal symptom symbol was divided by the total number of consonants. No other error analysis of consonant production was performed.

### 2.5 Statistical Analysis

Data were analysed using IMB SPSS Statistics version 30 for Windows. Descriptive statistics were illustrated using boxplots showing the median, interquartile range (IQR), minimum and maximum values (whiskers) and any potential outliers. A paired‐sample *t*‐test was performed on PCC between age groups, and group differences related to surgical protocol were assessed using one‐way ANOVA for PCC and percentage of oronasal symptoms at ages 3, 5 and 10. When no group differences were found, analyses were performed on the full sample.

Correlations between dichotomous early consonant variables and continuous outcomes at age 3, 5 and 10 were examined using Pearson's point biserial correlation. Predictive analyses were conducted using stepwise linear regression including the 18‐month variables and background factors. Statistical significance was set at *p* < 0.05.

## 3 Results

Below are results related to the research questions presented. At the end of the results section, separate information on a subgroup that performed low on PCC at 10 years is presented.

### 3.1 PCC and Oronasal Symptoms in Relation to Primary Surgical Protocol

**FIGURE 1:**
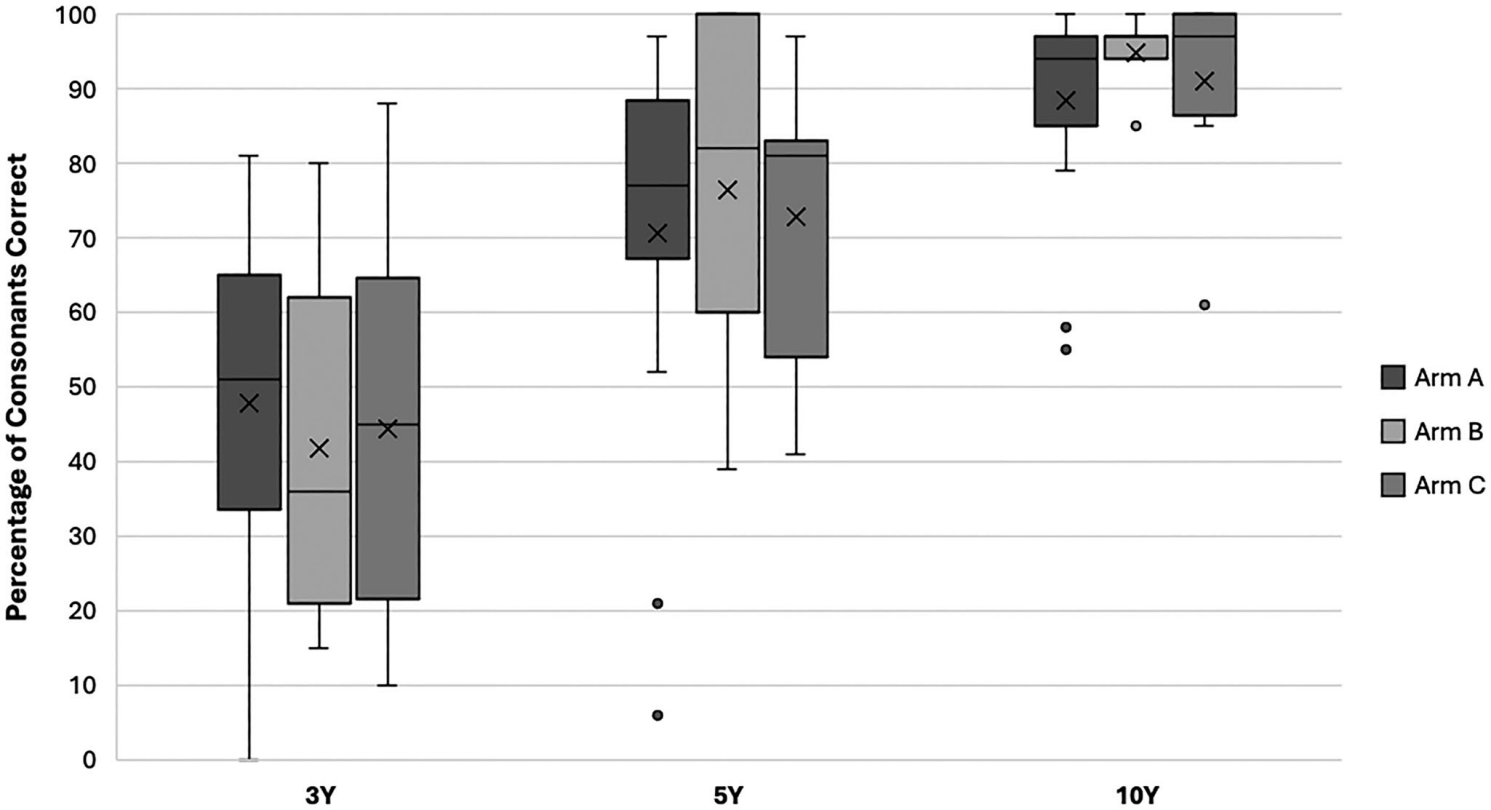
Longitudinal figures on percentage of consonants correct (PCC) at 3, 5 and 10 years of age in 32 children with UCLP treated according to different surgical protocols (Arm A: *n* = 16, B: *n* = 7, C: *n* = 9).

**FIGURE 2:**
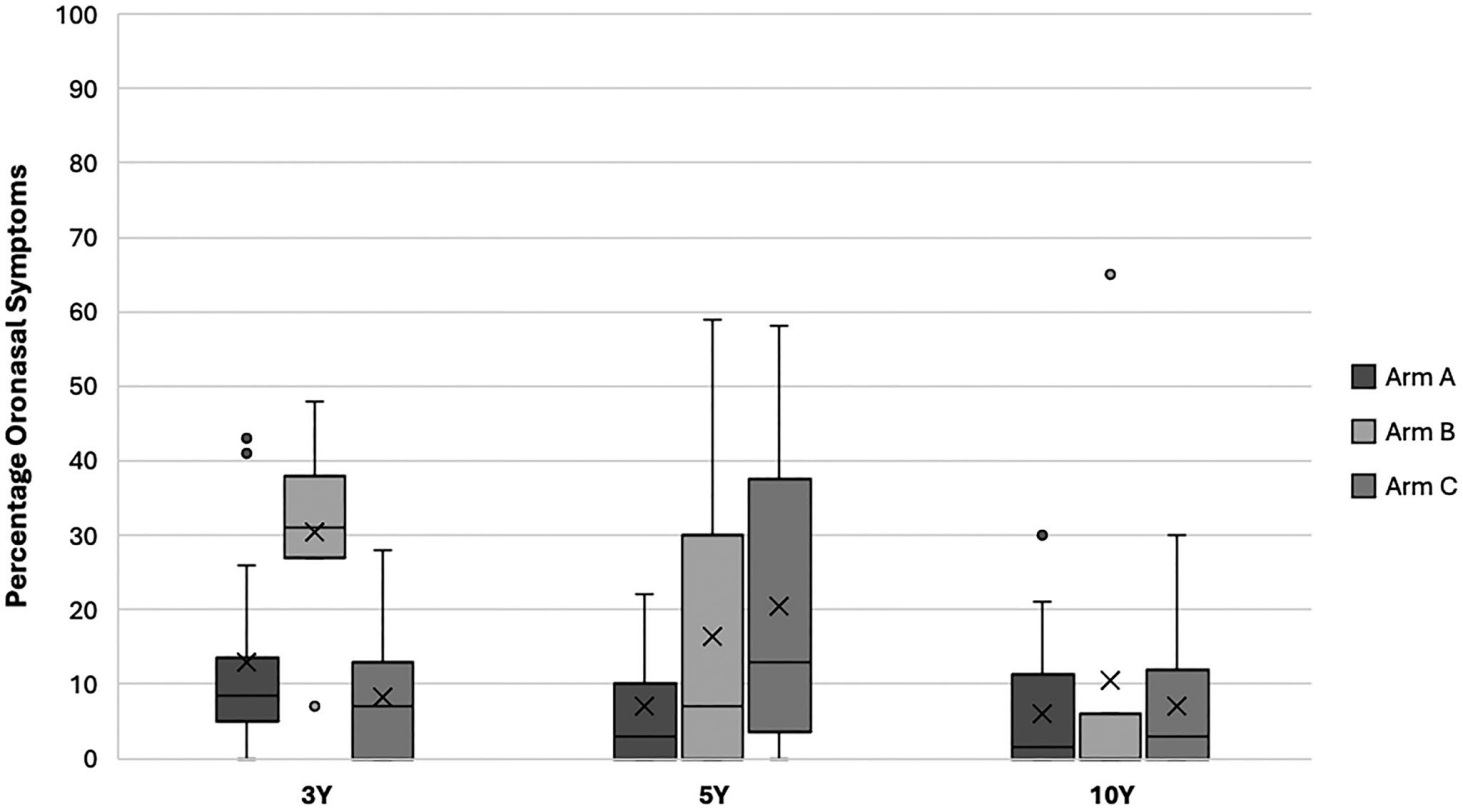
Longitudinal figures on percentage oronasal symptoms at 3, 5 and 10 years of age in 32 children with UCLP treated according to different surgical protocols (Arm A: *n* = 16, B: *n* = 7, C: *n* = 9).

There were no differences in PCC at 3 (*F* = 0.16, *p* = 0.851), 5 (*F* = 0.15, *p* = 0.858) and 10 (*F* = 0.70, *p* = 0.503) years of age across the three surgical protocols (Figure 1). However, the percentage of oronasal symptoms at 3 years of age was significantly higher in Arm B, where the hard palate remained unrepaired, compared to the other two surgical protocols (*F* = 7.68, *p* = 0.002) (Figure 2). No difference in oronasal symptoms at 5 (*F* = 2.43, *p* = 0.106) and 10 (*F* = 0.27, *p* = 0.765) years of age was related to surgical protocol.

Thus, the only surgery‐related difference observed was a higher prevalence of oronasal symptoms at 3 years of age in the group with an unrepaired hard palate. The remaining analyses are therefore based on the entire group.

### 3.2 PCC at Age 3, 5 and 10 Years

**FIGURE 3:**
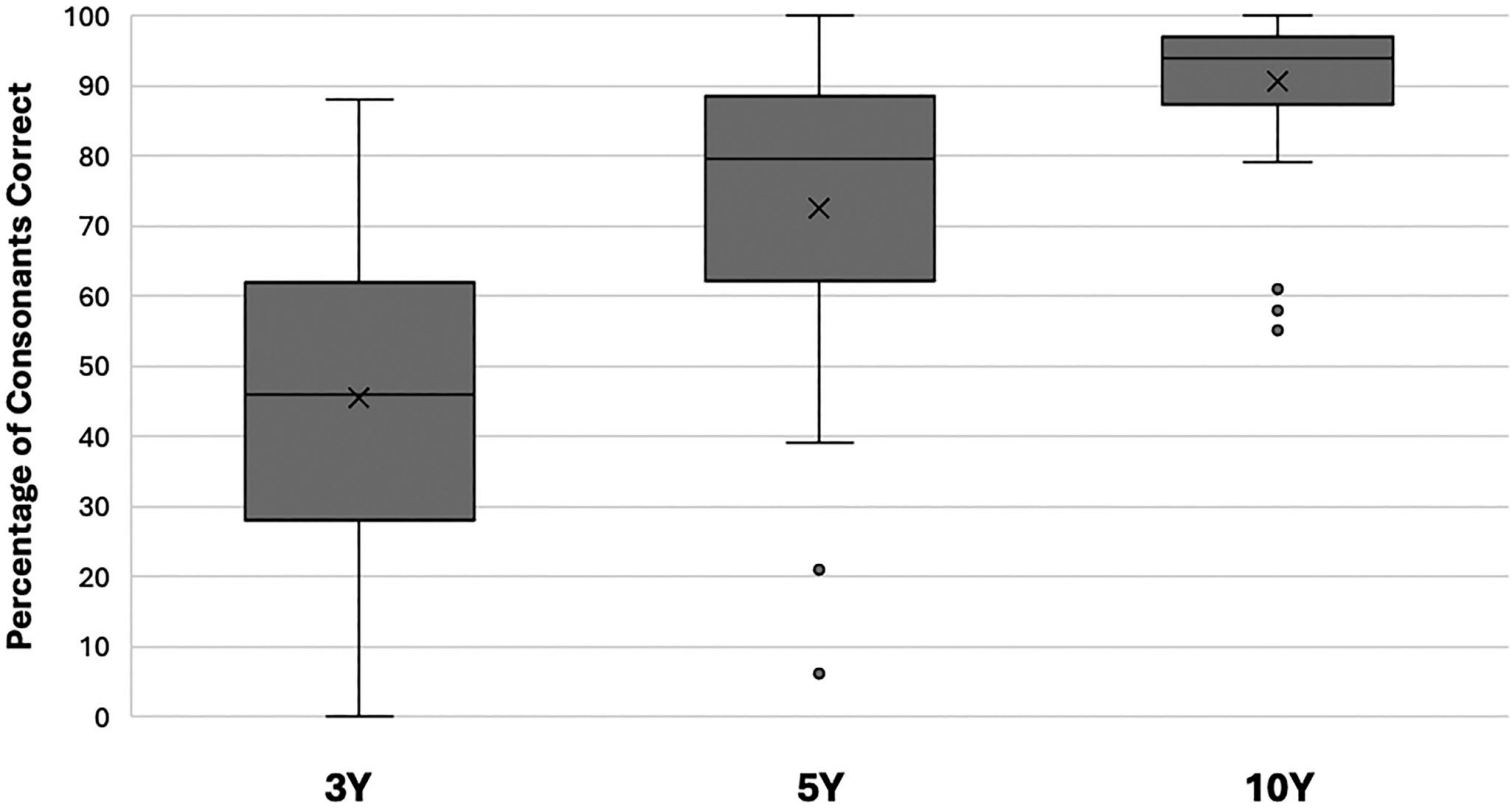
Longitudinal figures on percentage of consonants correct (PCC) at 3, 5 and 10 years of age in all 32 children with UCLP.

PCC increased with age, with median values of 46.0% at 3 years, 79.5% at 5 years and 94.0% at 10 years (Figure 3).

At Age 3, individual variability in PCC scores was considerable, as illustrated by the wide whiskers in the boxplot. Variation decreased slightly by Age 5, and by Age 10 most participants had relatively high PCC scores (Figure 3). Test of differences in mean (standard deviation, SD) between the age groups were statistically significant (Table 2).

**TABLE 2:** Group differences in PCC (mean, standard deviation [SD] confidence interval [CI]) between ages using a paired‐sample *t*‐test.

PCC (%)	Mean (SD)			CI	*t* (df 31)	*p*
3–5 years	45.5 (23.9)	72.5 (22.7)	−26.9 (20.1)	−34.171 to –19.704	−7.595	<0.001
5–10 years	72.5 (22.7)	90.5 (12.0)	−18.1 (16.2)	−23.914 to –12.211	−6.296	<0.001
3–10 years	45.5 (23.9)	90.5 (12.0)	−45.0 (22.1)	−52.982 to –37.018	−11.498	<0.001

### 3.3 Impact of Early Consonant Production on PCC

The presence of oral stops at 18 months was associated with higher PCC at all ages, and also dental/alveolar place of articulation at 5 and 10 years (Table 3). Additionally, the number of different consonants at 18 months was positively associated with PCC at Age 5 of but not at Ages 3 or 10.

**TABLE 3:** Pearson's correlation coefficient (*r*) with 95% confidence interval (CI),^a^ for the relationship between variables assessed at 18 months and the PCC at 3, 5 and 10 years in 32 children.

	PCC, 3 years		PCC, 5 years		PCC, 10 years	
Variables at 18 months	*r*	CI	*r*	CI	*r*	CI
Oral stops	0.40^*^	0.06, 0.66	0.66^***^	0.40, 0.82	0.60^***^	0.32, 0.78
Dental stops	0.33	−0.18, 0.61	0.46^**^	0.13, 0.70	0.41^*^	0.07, 0.66
Number of different consonants	0.29	−0.06, 0.58	0.43^*^	0.09, 0.68	0.14	−0.22, 0.47

^a^Bias adjusted estimation based on Fishers's r‐to‐z transformation.

^*^ *p *< 0.05.

^**^ *p* < 0.01.

^***^ *p* < 0.001.

**FIGURE 4:**
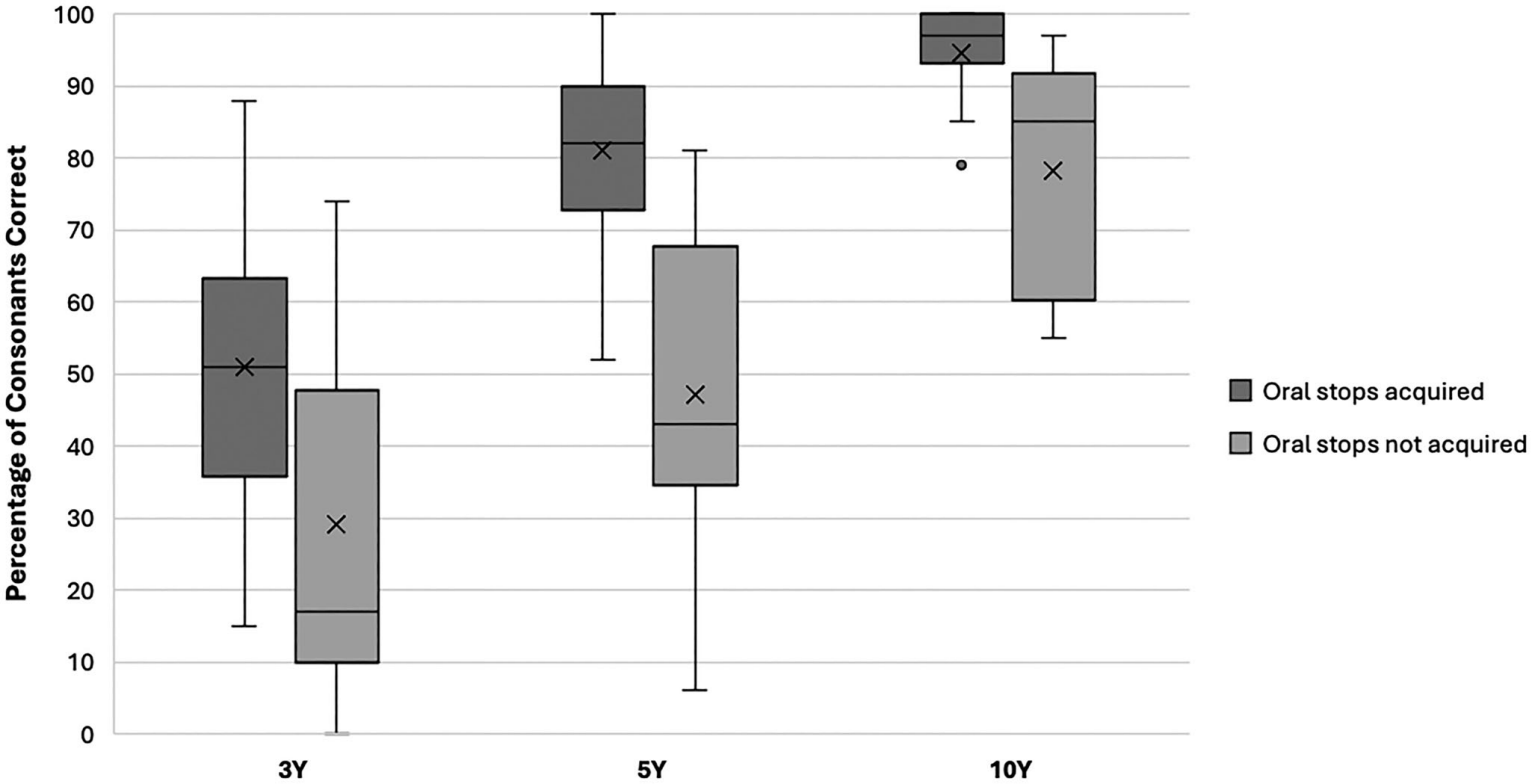
Longitudinal figures on PCC at 3, 5 and 10 years of age divided by presence of oral stops (*n* = 24) or not (*n* = 8) at 18 months in all 32 children with UCLP. An oral stops was considered acquired at 18 months if it was noticed twice or more in different utterances.

The clear distinction in PCC between children with and without oral stops at 18 months at all three ages is illustrated in Figure 4.

Logistic regression analysis confirmed that the presence of oral stops at 18 months significantly predicted higher PCC at all ages, whereas the other babbling variables did not.

Based on these models, the presence of oral stops at 18 months predicted PCC scores that were approximately 22% points higher at 3 years of age, 34% points higher at 5 years and 16% points higher at 10 years (Table 4).

**TABLE 4:** Prediction values from linear regression analysis of presence (yes/no) of oral stops at 18 months of age^a^ on level of PCC at 3, 5 and 10 years of age.

	Overall model		Oral stops		
Outcome measure	*R* ^2^	Adjusted	*p*	*B*	CI
PCC 3 years	0.16	0.13	0.022	21.875	3.338, 40.412
PCC 5 years	0.43	0.41	<0.001	33.958	19.427, 48.490
PCC 10 years	0.43	0.40	<0.001	18.429	9.806, 27.051

^a^A consonant was considered acquired if it was noticed twice or more in different utterances.

### 3.4 Oronasal Symptoms on Consonants at 3, 5 and 10 Years of Age

**FIGURE 5:**
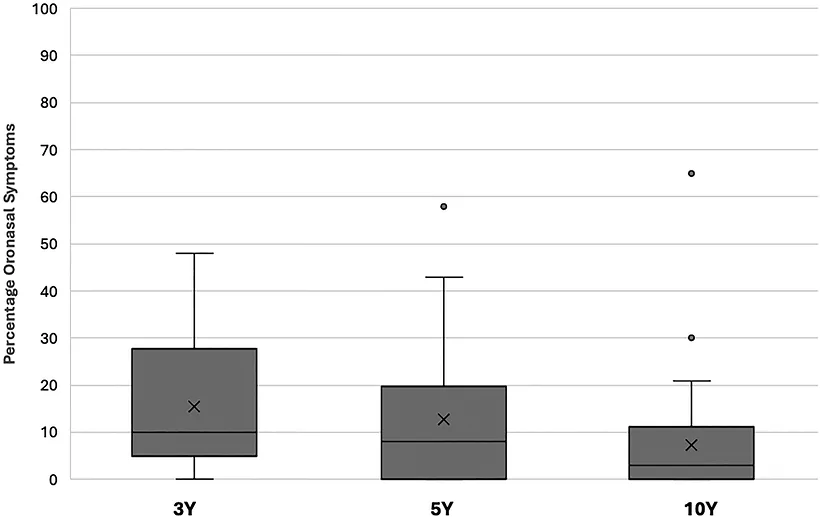
Longitudinal figures on percentage of oronasal symptoms at 3, 5 and 10 years of age in all 32 children with UCLP.

Oronasal symptoms decreased with age, with median values of 10.0% at 3 years, 8% at 5 years and 3% at 10 years (Figure 5).

At Age 3, individual variability in oronasal symptom scores was considerable, as illustrated by the wide whiskers in the boxplot. There were statistically significant lower percentages of oronasal symptoms at Age 10 compared to 3 and 5 years of age respectively, but not between 3 and 5 years of age (Table 5).

**TABLE 5:** Group differences in oronasal symptoms (mean, standard deviation [SD] confidence interval [CI]) between ages using paired sample *t*‐test.

Oronasal symptoms (%)	Mean (SD)			CI	*t* (df 31)	*p*
3–5 years	15.4 (14.1)	12.8 (16.2)	2.6 (17.9)	−3.825–9.075	0.830	0.413
5–10 years	12.8 (16.2)	7.3 (13.4)	5.5 (13.8)	0.508–10.492	2.247	0.032
3–10 years	15.4 (14.1)	7.3 (13.4)	8.1 (17.3)	1.887–14.363	2.656	0.012

### 3.5 Influence of Background Variables

There was a significant negative correlation between PCC at 10 years of age and having undergone secondary surgery up to 10 years (*r *= –0.557, *p *< 0.001). Regarding the 23 children (72%) who improved to peer level at Age 10, three had received secondary velopharyngeal surgery. Significant negative correlations were also found between PCC at 5 years and number of ST visits before Age 5 (*r *= –0.471, *p *= 0.007), and PCC at 10 years and number of speech‐therapy visits before Age 10 (*r *= –0.348, *p* = 0.05). These findings indicate that children who had poorer speech received more secondary surgery and more speech therapy, yet continued to have lower PCC scores than those who required no secondary surgery and fewer (or no) speech therapy visits. No clear associations were found between hearing levels in the better ear at 3 or 5 years of age and the speech outcomes at any age. There were no significant differences in outcomes between girls and boys.

### 3.6 Below Peer‐Level Subgroup at Age 10

The individual variability included slow and/or low improvement of PCC with a total of 9 (28%) children who did not reach peer level (≥91%; Lohmander, Lundeborg, et al. 2017) (Table 6). Out of these, PCC developed to a very low (in three) or rather low (in six) level at Age 10, one of these with a very low PCC‐score (39%) at Age 5. Five of the nine children had palatal surgery according to the common Arm (A), three to the local one‐stage protocol (Arm C) and one to the local two‐stage protocol (Arm B). Six of them received secondary velopharyngeal surgery between 5 and 10 years of age, and seven of them between 18 and 67 speech therapy visits. For three of them, the PCC‐score declined; at Age 5 in two, and at Age 10 in one.

**TABLE 6:** Information on the nine children who did not improve the percentage of consonants correct (PCC) to the level of peers at 10 years.^6^

PCC	ID	Arm^1^	Sec. Surg. < 10 Y	ST^2^ < 5 Y	Total ST^2^ ≤ 10 Y	PCC 3 Y %	PCC 5 Y %	PCC 10 Y %	OroNasal^3^ 3 Y %	OroNasal^3^ 5 Y %	OroNasal^3^ 10 Y %
Improved to very low level^4^	3	A	Yes	16	28	3	21	58	10	0	3
	10	A	Yes	22	67	0	6	55	0	3	15
	30	C	Yes	27	29	21	41	61	13	13	12
Improved but not to peer level^5^	22	B	Yes	14	38	14	39	85	38	15	6
	31	C	Yes	17	20	66	82	88	28	58	3
	26	C	Yes	15	22	87	80	85	13	43	30
	6	A	No	6	9	85	97	88	12	3	0
	7	A	No	13	18	74	72	85	26	19	12
	14	A	No	1	10	62	77	79	10	0	0

^1^A = lip and soft palate closure at 4 months, hard palate closure at 12 months (common Arm).

B = lip and soft palate closure at 4 months, hard palate closure at 3 years. NB: the hard palate cleft was unoperated at the 3‐year assessment.

C = lip closure at 3 months, soft and hard palate closure at 12 months.

^2^ST = Speech Therapy visits = proxy for speech intervention.

^3^OroNasal symptoms include phonetically transcribed audible nasal emission, snortings/velopharyngeal friction sounds, weak pressure on consonant and nasal realisation of oral consonant.

For example: 1 occurrence = 3%, 2 occurrences = 6%–7%, 3 occurrences = 10%, 4 occurrences = 12%–‐13%, 5 occurrences = 15%, 18 occurrences = 58%.

^4^10‐Year PCC is below normative data for 3‐year‐olds (Mean 82.0%, SD 14.26).

^5^10‐Year PCC is below normative data for 5‐year‐olds (Mean 96.3%, SD 5.44).

^6^Normative data for 10‐year‐olds (Mean 97.0%, SD 5.97).

The percentage of oronasal symptoms also varied among the nine children and across ages (Table 6). However, for three children with improvement to very low level, two increased the oronasal symptoms and for one child there was no change. The remaining six children, who all improved, also had a decrease in percentage of oronasal symptoms.

## 4 Discussion

The aim of this study was to investigate the longitudinal development of consonant proficiency in Swedish children with UCLP participating in the Scandcleft project and treated according to three different surgical protocols. In summary, despite large individual variation, the findings showed that consonant proficiency (PCC) improved with age regardless of surgical protocol, and that the acquisition of oral stops by 18 months was a strong predictor of later consonant proficiency, even up to 10 years of age. No significant differences between the surgical protocols were found with respect to percentage of oronasal symptoms except at 3 years. The significantly higher percentage of oronasal symptoms in Arm B compared with Arms A and C was most likely due to the hard palate remaining unrepaired at Age 3. At Ages 5 and 10 years, this difference had disappeared. These findings are consistent with the Scandcleft results at Age 5 (Willadsen et al., 2017; Lohmander, Persson, et al. 2017). At Age 10, some differences between Arms were reported in the Scandcleft project, but because measures of PCC and velopharyngeal function contradicted each other, no protocol could be deemed superior (Willadsen et al., 2023). The absence of significant protocol‐related differences in the present study suggests that surgical protocol alone cannot explain the large variability in PCC outcomes indicating that all three protocols are similarly effective with respect to speech outcomes. For this reason, subsequent analyses were conducted on the full sample.

### 4.1 PCC at Ages 3, 5 and 10 Years

Although few longitudinal studies of cleft palate speech exist, and even fewer have used PCC as an outcome measure, the available evidence aligns with the present findings: consonant proficiency in children with UCLP generally improves with age, albeit with individual exceptions. Within the main Scandcleft project, the longitudinal follow‐up at 5 and 10 years showed a clear average increase in PCC between these ages with a small number of individual declines (Persson et al., 2025). This pattern was also found in the present substudy of Swedish‐speaking participants, with 84.4% of the children improving at each test occasion, while 9.5% showed a decline between 3 and 5 years and 6.0% between 5 and 10 years. PCC scores in this study closely reflected those reported in the main Scandcleft analyses. The median PCC at 5 years in the Scandcleft project was 80% (Willadsen et al., 2017), compared with 79.5% in the present study. At 10 years, 65% of children in Scandcleft had reached a PCC mean score of ≥91% (peer level) (Willadsen et al., 2023), compared with 68.8% in the present study.

Outside the Scandcleft project, a Swedish intercentre study reported a median PCC score of 93.9% at Age 5 (Klintö et al., 2019), surpassing both the main Scandcleft and those of the current substudy (see above). At Age 10, 92.6% of the group in the intercentre study had achieved peer‐level PCC with a mean score of 98.4% (Havstam et al., 2023), exceeding the mean PCC scores in the main Scandcleft project with 86%–92% in the different Arms and in the present study (90.5%). These discrepancies might reflect differences in recording quality: Scandcleft recordings were collected over a 10‐year period of rapid transition from analogue to digital technology, introducing variability despite standardised training and procedures. The intercentre study, conducted nearly 20 years later, benefited from high‐quality recording experience gained from the Scandcleft project, which may partly explain the higher scores. Improved clinical care may also have contributed. At Age 3 years was recently reported a median PCC score of 38% based on data collected on a phrase sample by 15 English‐speaking children (Chee‐Williams et al., 2026). This is comparable to the somewhat higher median level (50%) for the measure adjusted to age and collected on single words, of 18 Swedish‐speaking 3‐year‐olds in a previous Scandcleft subproject (Klintö et al., 2014). The comparison median PCC score achieved by the English‐speaking peers without cleft was significantly higher (70%) even though somewhat lower than Swedish normative data for the age group (82%). The difference is presumably related to the speech material used where single words should be used if the aim is to collect data on the best production, whereas sentences is a relevant choice for connected speech (Klintö et al., 2011). Large ranges in both the cleft groups and the peer groups reveal a substantial individual variation at this early age. Furthermore, interreliable transcription may be challenging at this early age, which was revealed in one of the Scandcleft substudies, where the lower range value was 50% (Raud Westberg et al., 2019). Nevertheless, children with CP±L achieve lower PCC scores than their peers without cleft. This holds true also for the older ages. Klintö et al. (2016) found similar results at 5 years, and Lohmander and Persson (2008) at both 3 and 5 years. In the present study, PCC at all ages was below the normative values on SVANTE (Swedish Articulation and Nasality Test), including data on established consonant phonemes in Swedish children at different ages (Lohmander, Lundeborg, et al. 2017), though the gap narrowed with age. At 3 years, the mean PCC was approximately 2.5 SD below the normative value; by 5 years, around 1.5 SD below; and by 10 years, approximately 1 SD below. When outliers were removed, the gap was smaller: 1 SD at age 5 and 0.4 SD at age 10. This illustrates the effect of a small subset of children with markedly low scores on group means.

### 4.2 Comparative Analysis Between Early Consonant Production and PCC at Ages 3, 5 and 10 Years

This study showed that acquisition of oral stops by 18 months predicted higher PCC scores at all later ages, reinforcing previous research demonstrating that early development of oral stops is a strong indicator of later consonant proficiency. Chapman et al. (2003) showed that early stop consonant production and size of true consonant inventory at 13 months in children born with CLP were associated with the same measures in words at 21 months. The significance of oral stops was also shown by Klintö et al. (2014) and Raud Westberg et al. (2019). From the current study, it seems that this might apply even up to the age of 10. Earlier findings also show significant correlations between dental stops at 18 months and a high PCC at 3 and 5 years (Lohmander & Persson, 2008), and between anterior/dental/oral stops at 18 months and PCC at 3 years (Klintö et al., 2014; Lohmander et al., 2021). The present findings only partially support these earlier results, however. Dental placement was correlated with PCC at 5 and 10 years of age, but the effect was weak and did not independently predict PCC in regression analyses. While the number of different consonants has been identified as a predictor (e.g., Scherer et al., 2008; Raud Westberg et al., 2019), this was not the case in the present study, where a significant correlation was found only at Age 5. Differences in methodology might explain this; earlier studies used phonetic transcription, whereas the current study used observational data at 18 months, and validity for number of different consonants has been shown to be low (Lieberman & Lohmander, 2014).

### 4.3 Prevalence of Oronasal Symptoms at Ages 3, 5 and 10 Years

The percentage of oronasal symptoms steadily decreased on the group level with age. However, the difference between 3 and 5 years was not statistically significant. Thus, the residual cleft closure in Arm B seems not to have had a major impact on the presence of oronasal symptoms in the whole group. At Age 5, half of the group (16) still had oronasal symptoms, where more than two consonants were affected (corresponding to a percentage of oronasal symptoms of >7%). At 10 years of age, this prevalence was reduced to 28% (9) of the children. Four of the seven who improved had received secondary surgery.

This likely reflects VPC, in 72% at 10 years, even though the absence of hypernasality ratings in this study limits direct comparison with other measures of VPF, such as VPC‐Sum or VPC‐Rating. The results are nevertheless similar to main Scandcleft findings reporting VPC‐Sum, where 35%–61% of children had velopharyngeal competence at 5 years (Lohmander, Persson, et al. 2017) and 65%–80% at 10 years (Willadsen et al., 2023). The Swedish intercentre studies reported even higher rates (Klintö et al., 2019; Havstam et al., 2023), perhaps due to differences in assessment methods (VPC‐Rating vs. VPC‐Sum). Even though assessment with the two methods has been compared and found valid (Lohmander, Hagberg, et al. 2017), the VPC‐Sum is more sensitive. Another explanation could be improved VPF outcomes in later cohorts.

### 4.4 Impact of Background Variables

The primary surgical protocol was not associated with the main outcome. Furthermore, neither number of speech therapy visits nor all secondary surgery was associated with achieving peer‐level PCC (only three out of nine). The majority of children who received these interventions had poor speech both before and after treatment, suggesting that while interventions are correctly directed toward children with greater needs, they do not eliminate the underlying speech difficulties. However, only the number of ST visits was used in the analysis. The original plan was to report the type of ST but was unfortunately not possible to collect in the main Scandcleft project due to the very different circumstances or settings, cultural differences and SLP/T different backgrounds. Another possible factor, not investigated in the present study, is surgical experience. However, in the main Scandcleft project was stated that the results ‘indicated that surgery was more problematic for surgeons who were still gaining experience with an unfamiliar surgical protocol’ (Rautio et al., 2017). Furthermore, has cleft width, based on cleft type, been shown to predict consonant proficiency (Okhiria et al., 2026). Early hearing impairment at 18 and 30 months has been shown to relate to poorer PCC at 3 years (Lohmander et al., 2021). At later ages, no such associations have to our knowledge been reported. However, it has been speculated that the rather common difficulties with sibilants may be related to high‐frequency hearing loss among older children, adolescents and young adults with CLP (Flynn & Lohmander, 2014).

### 4.5 Individual‐Level Considerations

There is no clear explanation of the poor results in the group of nine children; no clear association with primary surgical protocol, presence of secondary surgery or speech therapy visits. The individual variation may be related to unknown additional conditions, such as language disorder (LD) or neurodevelopmental disorder (NDD), proven more common in individuals with clefts compared to peers without clefts (Sparks Lancaster et al., 2020; Tillman et al., 2018), which may influence outcomes and should be thoroughly reviewed and considered in future studies. Clinicians should preferably be prepared for the presumed large variation in the population of children with clefts and, in addition to standard documentation and assessment related to the structural defect, add individualised assessment of speech, language and communicative interaction.

### 4.6 Limitations

This study included a relatively small sample, and group sizes differed by surgical protocol, limiting statistical power. Observational assessment at 18 months had low validity for the number of different consonants (Lieberman & Lohmander, 2014), and inter‐transcriber agreement at Age 3 was variable. The absence of hypernasality ratings required reliance on oronasal symptoms as an outcome measure for VPF, while a more detailed and valid measure would have been VPC‐Sum. However, the pattern of results closely mirrored the main Scandcleft findings at 5 and 10 years of age, suggesting that the sample size was representative. A notable limitation is that outliers affected mean PCC values, though median and interquartile range were reported to mitigate this. As early hearing impairment has been shown to relate to poorer PCC at 3 years, the lack of data on hearing prior to that age is a limitation. In addition to factors possibly related to individual variation (see above), adding socioeconomic status may also be relevant. Finally, it cannot be excluded that the speech deviances could have impacted the intelligibility. However, since intelligibility was not assessed in the main Scandcleft project, it could not be considered in the present study.

## 5 Conclusions and Implications

The findings indicate that early consonant production, particularly the presence of oral stops at 18 months, is strongly associated with and predictive of consonant proficiency throughout childhood. This supports existing evidence that early consonant acquisition forms a critical basis for later language development. At the same time, the substantial individual variation highlights the need to complement group‐level analyses with individual‐level data, particularly in longitudinal studies, to capture diverse developmental trajectories and identify early markers of later difficulties.

It is essential to identify children at risk early, using markers such as the absence of oral stops, low PCC, or atypical errors at key developmental ages. Parent‐implemented early intervention strategies (Scherer & Kaiser, 2007; Hardin‐Jones & Chapman, 2008; Finestack et al., 2022) may help promote speech‐language development and support school readiness. Beyond optimising surgical techniques and speech therapy approaches, cleft teams should prioritise identifying risk factors for persistent speech difficulties or unexpected negative speech development. Early hearing assessment and management, ongoing monitoring of speech development, and consideration of co‐occurring developmental language difficulties are thus recommended.

## Ethics Statement

The Scandcleft study was approved by the Regional Ethical Committee in Gothenburg (R257‐97) and in Stockholm (Dnr. 97–372). All parents gave written informed consent for participation.

## Conflicts of Interest

The authors declare no conflicts of interest.

## Data Availability

Data in this substudy are part of a multicenterstudy and not available apart from by the included researchers.

## 1

Brief information on studies on longitudinal early development of consonant and speech production and on longitudinal speech outcome at preschool age, school ages and young adulthood. The order of the studies is based on number of ages in the data analysis beginning at babbling level, then the ones beginning at 3–5 years.

**Table:**

1st Author, year	Country	No of children	Cleft type	Comparisongroup	Ages at data collection	Relevant findings
Jones, 2003	USA	14	UCLP BCLP	14 NonCleft	11 m^6^, 17 m	11 m preop vs. 17 m postop: Production of oral stops doubled. 17 m: Group differences—CLP had a lower amount of oral stops and alveolars. No difference in canonical syllables, consonant inventory size.
Willadsen, 2006^7^ Willadsen, 2013,^5^	Denmark	34	UCLP	35 NonCleft	11 m^7^, 18 m^8^	11 m: Consonant classes described in 2006. 18 m: Group differences in and correlation with word‐initial consonant (selectivity) at 11 m.
Lohmander, 2011	Sweden	9 18	UCLP	21 NCLP, TD^1^	12 m^7^, 18 m^8^	12 m: All had canonical babbling (CB). Correlation between mild hearing loss and fewer different consonants. 18 m: Group differences with lower frequency of oral stops and dentals.
Wilder, 2026^4^	USA	88	CP±L^3^	—	16 m, 24 m	16 m: Number of stop consonant production, reported vocabulary understood. 24 m: Predicted expressive vocab. size and M3L.
Hutters, 2001	Denmark	13	13 UCLP 2 BCLP	17 NonCleft	12 m^6^, 3 y	12 m: Group differences with higher occurrence of nonoral consonants in cleft group. 3 y: Group differences with lower number of correct word production in cleft group.
Klintö, 2014	Sweden	18	UCLP	20 NCLP, TD	18 m, 3 y	18 m: Number of oral consonants, stops and dental place. 3 y: Group differences regarding number of established phonemes and phonol processes Consonant variables correlated with PCC at Age 3.
Scherer, 2008	USA	13	CP±L	13 NCLP, TD^1^	12 m, 30 m	12 m: Group differences in Mean Babbling Level (6 m: Group differences in babbling frequency). 30 m: Group differences in and correlation with consonant inventory size and vocabulary production.
Chapman, 2003	USA	15	11 UCLP 4 BCLP	15 NonCleft	9 m^6^, 13 m, 21 m	9 m preop and 13 m postop: Group differences with NonCleft. Stop production and size of consonant inventory, respectively. 21 m: Correlated with vocabulary development and speech production.
Raud Westberg, 2019	Sweden	33	UCLP	—	12 m^6,7^, 18 m, 3 y	12 m: All but one had CB, higher occurrence of oral stops and anterior place in two‐stage group. 18 m: No group difference in consonant production. 3 y: No group difference PCC, correlation trend with early number of different consonants.
Lohmander, 2021	Sweden	15	11 CPO 4 UCLP	15 OME‐HI^2^	10 m, 12 m, 18 m, 3 y	10 m: Both groups low prevalence of CB (CP 54%, OME‐HL 77%). 12 & 18 m: Group differences on all consonant variables (oral stops, dental place, number of diff. cons.). 3 y: Both groups lower PCC compared to peers. Correlated with consonant variables, but not with CB. Hearing at 18 and 30 m explained 40% of variation.
Lohmander, 2008	Sweden	20	UCLP	9 NonCleft (NC)	18 m^7^, 3 y^7^, 5 y, 7 y	18 m: Group differences in the number of different consonants, lower‐frequency dental place. 3 y: Group differences in PCC, PCP and correlation with the number of different consonants. 5 y: Group differences in PCC, PCP and correlation with high frequency of dental stops. 7 y: Group differences in PCC, percentage of correct places (PCP).
Klintö, 2016	Sweden	29	UCLP	20 NCLP, TD^1^	3 y, 5 y^9^	3 & 5 y: Group differences cleft group and TD with lower PCC and more phonological processes. Within the cleft group, best PCC after complete palatal repair at 12 m.
Nyberg, 2014	Sweden	69	UCLP	—	5 y, 10 y	High occurrence of articulation deviances at 5 years of age, markedly reduced at 10 years. Included 43% secondary velopharyngeal surgeries, 7% velopharyngeal insufficiency at 10 y.
Persson, 2025	Sweden, Finland, Denmark, Norway, United Kingdom	320	UCLP	—	5 y, 10 y	Low speech proficiency at 5 y (23% at peer level), improved to10 y (56% at peer level). Included 34% secondary velopharyngeal surgeries. Speech proficiency at 5 y best predictor of speech proficiency at 10 y. Girls performed better throughout. No obvious evidence to recommend any of the protocol as superior.
Lohmander, 2012	Sweden	55	UCLP	—	5 y^8^, 7 y^8^, 10 y, 16 y, 19 y	High prevalence of oral consonant errors at 5 y (39%), at 7 y (23%), at 10 y (6%), at 16 and 19 y two and one individual. Included 11% secondary velopharyngeal surgeries. 8% and 9% had velopharyngeal insufficiency at 16 and 19 y.

*Note*: All correlations were positive.

^1^NCLP, TD = NonCleft Typically developing.

^2^OME‐HI = Otitis Media with Effusion related mild‐moderate Hearing Impairment.

^3^Soft palate cleft (14.4%), Hard and soft palate cleft (33%), Unilateral cleft lip and palate (37.5%), Bilateral cleft lip and palate (14.8%). Palatal surgery mean age 12 m (8.4–16.0).

^4^Mean utterance length of the three longest (M3L) and parent‐reported expressive vocabulary (CDI).

^5^Lexical selectivity was investigated, that is, consonant match at 11 months in words at 18 months.

^6^Unrepaired cleft palate.

^7^Repaired soft palate (4–6 m) and unrepaired hard palate.

^8^Half group repaired soft palate (4–6 m) and hard palate (12 m), half group repaired soft palate (4–6 m) and unrepaired hard palate.

^9^1/3 group repaired soft palate (4–6 m) and hard palate (12 m), 1/3 group repaired soft palate (4–6 m) and hard palate (36 m, after 3 y assessment), 1/3 complete cleft palate at 12 m.
